# Up-Regulation of NDRG2 in Senescent Lens Epithelial Cells Contributes to Age-Related Cataract in Human

**DOI:** 10.1371/journal.pone.0026102

**Published:** 2011-10-17

**Authors:** Zi-Feng Zhang, Jian Zhang, Yan-Nian Hui, Min-Hua Zheng, Xin-Ping Liu, Peter F. Kador, Yu-Sheng Wang, Li-Bo Yao, Jian Zhou

**Affiliations:** 1 Department of Ophthalmology, Eye Institute of Chinese PLA, Xi-Jing Hospital, Fourth Military Medical University, Xi'an, China; 2 Department of Biochemistry and Molecular Biology, State Key Laboratory of Cancer Biology, Fourth Military Medical University, Xi'an, China; 3 Department of Medical Genetics and Developmental Biology, State Key Laboratory of Cancer Biology, Fourth Military Medical University, Xi'an, China; 4 Department of Pharmaceutical Sciences, College of Pharmacy, University of Nebraska Medical Center, Omaha, Nebraska, United States of America; 5 Department of Ophthalmology, School of Medicine, University of Nebraska Medical Center, Omaha, Nebraska, United States of America; Instituto Butantan, Brazil

## Abstract

**Background:**

Human N-Myc downstream regulated gene2 (NDRG2), a novel gene has been cloned and shown to be related to a number of cellular processes, including proliferation, differentiation, stress, and apoptosis. NDRG2 has also been linked to age-related Alzheimer's disease. Since the role of this gene in senescence is limited, we have investigated the potential role of NDRG2 in human lens epithelial cells (HLECs), a paradigm implicated in age-related cataract.

**Methodology/Principal Findings:**

Cultured HLECs (SRA01/04) were subjected to prolonged exposure to low dose of H_2_O_2_ to simulate senescence. After being exposed to 50 µM H_2_O_2_ for 2 weeks, HLECs senescent-morphological changes appeared, cell viability decreased dramatically, cell proliferation reduced from 37.4% to 16.1%, and senescence-associated β-galactosidase activity increased from 0 to 90.3%. Ndrg2 protein expression was also significantly increased in these senescent cells. To induce overexpression of NDRG2, SRA01/04 cells were infected with the adenoviral vector of NDRG2. In these cells, overexpression of NDRG2 resulted in a fibroblast-like appearance and the cell viability decreased about 20%. In addition, the NDRG2-overexpression cells demonstrated 20% lower viability when exposed to 50–200 µM H_2_O_2_ for acute oxidative stress. Furthermore, the expression of NDRG2 from age-related cataracts was up-regulated 2-fold at both mRNA and protein levels compared with the clear lenses.

**Conclusions/Significance:**

NDRG2 is up regulated not only in the ageing process of HLECs *in vitro* but also in the cells from human age-related cortical cataract *in vivo*. Up-regulation of NDRG2 induces cell morphological changes, reduces cell viability, and especially lowers cellular resistance to oxidative stress. NDRG2-mediated affects in HLECs may associate with age-related cataract formation.

## Introduction

N-Myc downstream-regulated gene 2 (NDRG2) [1], [2], [3] is one of the four members of the N-Myc downstream-regulated gene (NDRG) family, a new class of Myc-repressed genes composed of NDRG1–4 [1], [4], [5], [6], [7], [8], [9], [10]. The NDRGs are highly conserved in plants, invertebrates and mammals, suggesting that the NDRG family has important cellular functions [9]. Significant attention has been paid to this gene family due to its potential role as a tumor suppressor as well as its involvement in other diseases. Human NDRG2 (also named SYLD/KIAA1248), is located on chromosome 14q11.2, and was first cloned from a normal human brain cDNA library by subtractive hybridization [3]. NDRG2 has been proposed to be a candidate tumor suppressor gene, and its expression has been confirmed to be reduced or absent in many tumors [3], [11], [12], [13], [14], [15], [16], [17]. Inhibition of cell proliferation by overexpression of NDRG2 in malignant cancer cells has also been reported [18], [19], [20]. Accumulating data also suggests that NDRG2 regulates cellular differentiation and development *in vitro* and *in vivo* [21], [22], [23], [24], [25], and is involved in cellular response to stress [19], [26].

The NDRG family has been shown to be widely expressed in the nervous system [27], with brain tissue the most abundantly expressing NDRG2 [2], [3]. The critical function of NDRG2 in the nervous system was confirmed by its up-regulated expression in the brains of patients with Alzheimer's disease [28]. Expression of NDRG2 in affected brains is present in cortical pyramidal neurons, senile plaques and cellular process of dystrophic neurons. Since Alzheimer's disease is age-related, NDRG2 may also be associated with ageing. Although great progresses have been made in understanding the role of NDRG2, little is known of its function in ageing.

Age-related cataract, the leading cause of world blindness, is another major age-related disease [29], [30], [31]. The lens epithelium as a morphological entity in the human lens is first recognizable in the 5th–6th week of gestation. Nuclei are primarily present in epithelial cells and these metabolically acting cells are essential for the growth, differentiation and homeostasis of the lens [32]. The cells stay in this morphological state as the anterior epithelium of the lens for the rest of the life, making them an attractive paradigm for the study of the effects of ageing [33]. It is also the first lens cell layer that is exposed to aqueous-associated changes due to ageing. Therefore lens epithelial cells are ideally suited for investigating the role of NDRG2 in ageing.

In the present study, using prolonged exposure of human lens epithelial cells (HLECs) to low doses of H_2_O_2_ as a model of lens ageing, here we investigated the up-regulation of Ndrg2 protein in these cells. The overexpression of NDRG2 in HLECs resulted in fibroblast-like morphology changes, decreasing cell viability and resistance to oxidative stress. In addition, we also found expression of NDRG2 in HLECs from age-related cataracts was higher than clear lenses at both mRNA and protein levels.

## Results

### NDRG2 up-regulation in HLECs prolonged exposure to H_2_O_2_ *in vitro*

In order to determine whether NDRG2 is involved in the cellular senescence, we exposed cultured HLECs (SRA01/04) to prolonged low dose of H_2_O_2_ to simulate oxidative stress associate with ageing.

**Figure 1:**
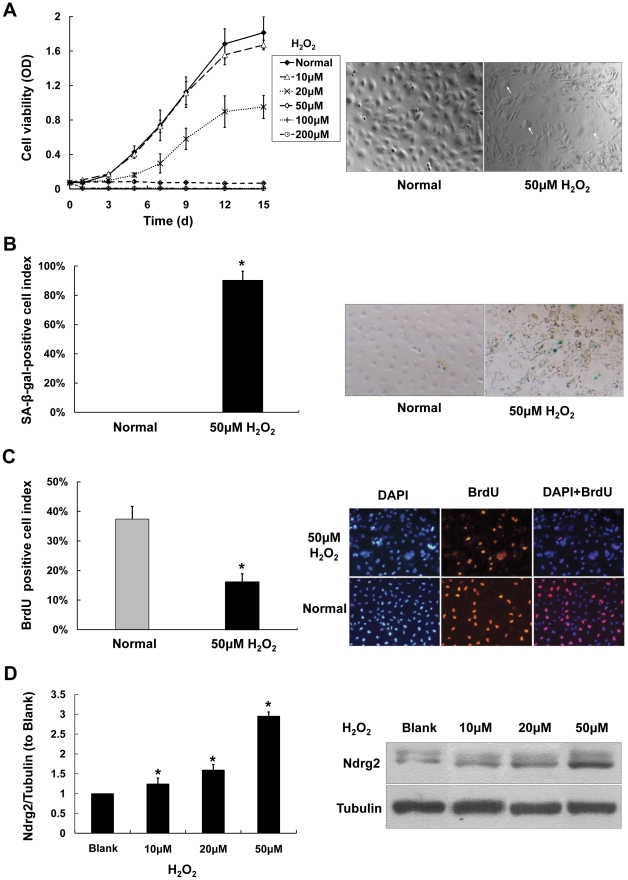
Characteristics induced by 2 weeks exposure of SRA01/04 cells to low-doses of H_2_O_2_. A. Shows that cell viability (Left) of SRA01/04 cells decreases with increasing concentrations of H_2_O_2_ as measured by the MTT assay (Mean± SD; n = 5.). Morphologic changes (Right) of SRA01/04 cells after 2 weeks exposure to 50 µM H_2_O_2_ demonstrate gross enlargement, flattening, and the accumulation of granular cytoplasmic inclusions (Arrows indicate the nuclei of typical senescent cells, 100×). B. Shows staining for SA-β-gal activity increased in SRA01/04 cells exposed to 50 µM H_2_O_2_ for 2 weeks expressed as either SA-β-gal-positive cell index (Mean± SD; n = 3. * *P*<0.05. Left) or actual cellular staining (Right). C. Shows that DNA synthesis decreased in SRA01/04 cells exposed to 50 µM H_2_O_2_ for 2 weeks. BrdU labeling in SRA01/04 cells expressed either by BrdU incorporation and DAPI cell viability labeling (Mean± SD; n = 3. * *P*<0.05. Left) or expression of BrdU labeling of cells (Right). D. Shows that Ndrg2 protein is up-regulated in SRA01/04 cells exposed to 0–50 µM H_2_O_2_ for 2 weeks (Mean± SD; n = 3. * *P*<0.05 compare with normal SRA01/04 cells).

H_2_O_2_ is cytotoxic at high concentrations. The viability of SRA01/04 cells exposed to various concentrations of H_2_O_2_ was investigated. Cell viability assay and morphological observation indicated that 10 µM of H_2_O_2_ had essentially no adverse effects on cell survival. With 20 µM H_2_O_2_, the growth curve declined and the doubling time became prolonged compared with untreated control cells, while cell morphology appeared normal. With exposure to 50 µM H_2_O_2_, the cell growth was arrested and maintained at this level for 2 weeks, which resulted in essentially a flatten growth curve with only a slight increase within the first 5 days (Fig. 1 A, left).

Morphology is an important criterion for judging cell senescence, because senescent cells become enlarged and demonstrate reduced saturation density. Morphological changes in SAR01/04 cells began to appear 3 days after exposure to 50 µM H_2_O_2_. Two weeks later, the morphology of the treated cells resembled that of senescent cell: with gross enlargement, flattening, and the accumulation of granular cytoplasmic inclusions (Fig. 1 A, right). At the dose of 100 and 200 µM, the cell viability dropped sharply immediately after initial exposure. The cells appeared “withered”, the cell body shrank and elongated, and granular inclusions aggregated in the cytoplasm after three days. Within 5–10 days, the cells detached and died. Therefore 50 µM H_2_O_2_ was chosen for the remaining experiments since replication of SRA01/04 cells was arrested and the cells displayed senescent-like morphological features. The following experiments were employed to confirm that these cells simulated ageing.

Senescence-associated β-galactosidase (SA-β-gal) staining has been used as an *in vitro* biomarker for cellular senescence [34], because blue β-galactosidase staining at pH 6.0 increases in senescent cells. In SRA01/04 cells exposed to 50 µM H_2_O_2_ for 2 weeks, the proportion of SA-β-gal staining cells was 90.3%±6.2%, but in normal SRA01/04 cells, the SA-β-gal staining was absent (Fig. 1 B).

A 5-Bromo-2′-deoxy-uridine (BrdU) incorporation assay was used to investigate DNA synthesis in cells. As shown in Fig. 1 C, BrdU incorporation was dramatically decreased from 37.4%±4.3% in the normal cells to 16.1%±2.8% in those exposed to 50 µM H_2_O_2_ for 2 weeks. This indicated that cell proliferation was inhibited by 50 µM H_2_O_2_.

In the remaining adherent cells after exposure to 50 µM H_2_O_2_, apoptotic cells were identified by terminal deoxynucleotidyl transferase-mediated dUTP nick-end labeling (TUNEL) assay. However, no significant difference was observed between H_2_O_2_ exposed and control cells (data not shown).

From the data above, one can conclude that after prolonged exposure to 50 µM H_2_O_2_, many changes took place in SRA01/04 cells. Not only senescent-like morphological changes but also other characteristics related to senescent cells, such as activation of SA-β-gal activity, the cessation of growth, inhibition of DNA synthesis, and the arrest of proliferation. Therefore these cells may mimic the ageing lens epithelial cells *in vivo*.

Exposure of SRA01/04 cells to increasing concentration of H_2_O_2_ (0 to 50 µM) also resulted in the up-regulation of NDRG2 in a dose-dependent manner (Fig. 1 D).

### Functional changes of HLECs induced by overexpression of NDRG2

**Figure 2:**
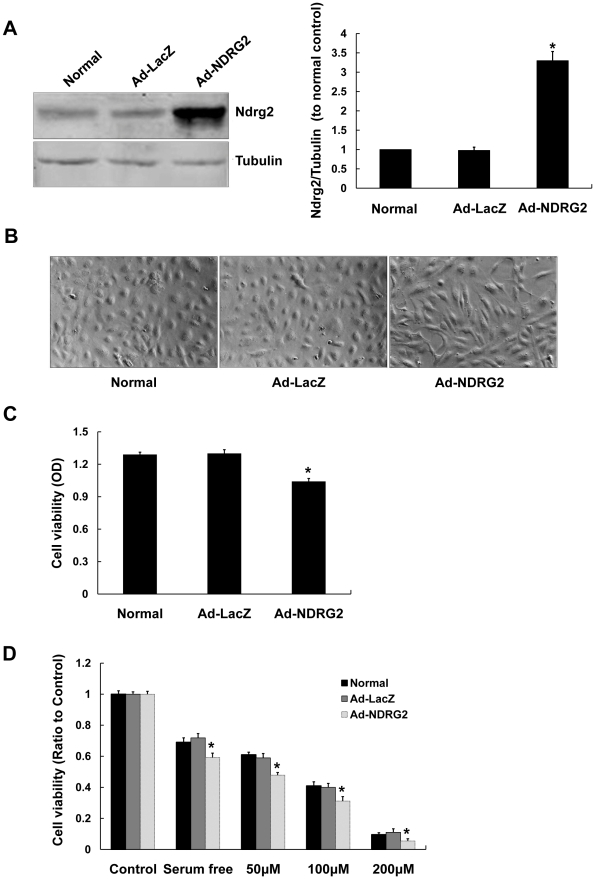
Functional changes of SRA01/04 cells infected with Ad-NDRG2. A. Shows by Western blot that protein expression of Ndrg2 in SRA01/04 cells is induced when cells are infected with Ad-NDRG2 but not Ad-LacZ (Mean± SD; n = 3. * *P*<0.05 compare with normal SRA01/04 cells). B. Shows that SRA01/04 cells infected with Ad-NDRG2 after 48 h show morphological changes that include fibroblast-like appearances (100×). C. Shows that cell viability in SRA01/04 cells decreased when infected for 48 h with Ad-NDRG2, but not Ad-LacZ. Determined by the MTT assay (Mean± SD; n = 5. * *P*<0.05). D. Shows cell viability (normalized to control cells) of uninfected and 48-hour infected SRA01/04 cells as determined by the MTT assay. These results suggest that SRA01/04 cells were more susceptible to oxidative stress after Ad-NDRG2 infection (Mean± SD; n = 5. * *P*<0.05 compare with uninfected and Ad-LacZ infected SRA01/04 cells).

To investigate the role of NDRG2 up-regulation, we infected SRA01/04 cells with the adenoviral vector of NDRG2 (Ad-NDRG2). Western blot analysis revealed that the expression of Ndrg2 protein in Ad-NDRG2-infected cells markedly increased by 48 h after infection (Fig. 2 A). Morphological changes observed in the NDRG2-infected cells included an initial cellular stretching, followed by a fibroblast-like appearance 12 h after infection. By 48 h, almost all cells displayed a fibroblast-like appearance (Fig. 2 B). Cell viability, measured by Methyl thiazolyl tetrazolium (MTT) colorimetric assay, decreased about 20% compared with Ad-LacZ infected control cells, while there was no difference between the Ad-LacZ infected and normal SRA01/04 cells (Fig. 2 C).

Since environmental factors such as oxidative stress have been implicated in the formation of age-related cataract, and H_2_O_2_ is the major oxidant involved in cataract formation [31], [35], we observed the response of NDRG2-infected cells to various concentrations of H_2_O_2_. After 24-hour exposure of H_2_O_2_, the cell viability decreased in both NDRG2-infected and control cells with increasing concentrations of H_2_O_2_ from 50 to 200 µM, but in NDRG2-infected cells it was about 20% lower than both normal and Ad-LacZ infected control cells (Fig. 2 D).This implied that overexpression of NDRG2 increased the sensitivity of SRA01/04 cells to oxidative injury.

### Differential expression of NDRG2 in HLECs between age-related cortical cataract and the clear lenses

In order to determine whether there are differential expressions of NDRG2 between age-related cataract and normal lenses, we examined NDRG2 in HLECs adhering to anterior capsules of lenses at both mRNA and protein levels. Anterior capsules from 30 clear lenses (mean age of 46.2±7.3, range 34–63 years) and from 90 lenses with age-related cortical cataracts (mean age of 65.3±8.7, range 47–90 years) were used for total RNA extraction and semi-quantitative RT-PCR study. 24 anterior capsules from clear lenses (mean age of 47.8±7.7, range 32–65 years) and 60 from cataractous lenses (mean age of 66.5±8.2, range 48–89 years) were used for western blot analysis. In addition, 5 anterior capsules from clear lenses (mean age of 45.7±6.4, range 36–57 years) and 10 from cataractous lenses (mean age of 68.7±9.3, range 57–86 years) were used for immunofluorescence staining (Table 1).

**Table 1:** Characteristics of specimen donors in this study.

Experimental methods	Group	Number	Age(Mean±SD)	Gender (Female/male)
RT-PCR	Clear	30	46.2±7.3	13/17
	Cataract	90	65.3±8.7	43/47
Western blot	Clear	24	47.8±7.7	11/13
	Cataract	60	66.5±8.2	31/29
Immunofluorescence	Clear	5	45.7±6.4	2/3
	Cataract	10	68.7±9.3	5/5

**Figure 3:**
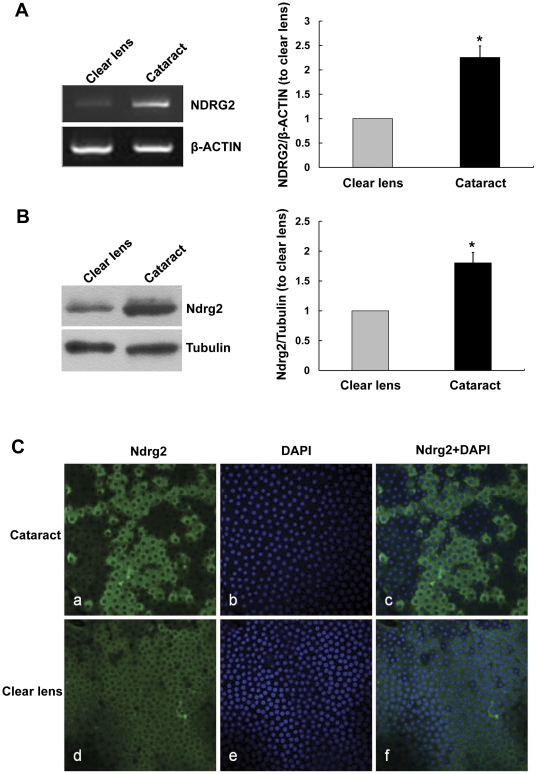
Differential expression of NDRG2 between HLECs from age-related cortical cataracts and clear lenses. A. Shows that NDRG2 by RT-PCR analysis is higher in HLECs from age-related cortical cataracts (65.3±8.7 years) versus clear lenses (46.2±7.3 years). (Mean± SD; n = 3. * *P*<0.05). B. Differential expression of Ndrg2 protein in HLECs from age-related cortical cataracts (66.5±8.2 years) and clear lenses (47.8±7.7 years). Measured by Western blots (Mean± SD; n = 3. * *P*<0.05). C. Whole-mount immunofluorescence staining of Ndrg2 (green) in anterior capsules from age-related cortical cataracts (a, b, c) and clear lenses (d, e, f). HLECs were visualized by DAPI staining (blue). Images from both groups were captured by confocal microscopy under the same exposure condition. The fluorescence of Ndrg2 protein, which is localized in the cytoplasm, was higher in the cataractous compared with clear lenses (400×).

For semi-quantitative RT-PCR analysis, total RNA from the cataract and clear lenses was extracted and reverse-transcribed respectively. Then, the presence of mRNA coding for NDRG2 and ACTIN was monitored by semi-quantitative PCR. PCR products, stained with Ethidium bromide, showed two bands with the expected size as 310 bp for NDRG2 and 602 bp for ACTIN (Fig. 3 A, left). ACTIN was expressed at similar levels in both groups. After normalizing the bands of NRDG2 to ACTIN, the relative expression of NDRG2 in the HLECs increased 2.26 folds in cataractous lenses compared with the clear lenses (*P*<0.05, Fig. 3 A, right).

Next Western blots were performed to investigate the differential expression of NDRG2 at the protein level between cataractous and clear lenses. Anterior capsules from 20 cataractous and 8 clear lenses were processed for total protein extraction, respectively. Bands at around 41 kDa, reacted with anti-Ndrg2 goat polyclonal antibody were detected in both the cataracts and clear lenses (Fig. 3 B, left). After normalizing the Ndrg2 to Tubulin bands, the relative expression of Ndrg2 protein significantly increased to 1.80 folds in cataract lenses compared with in the clear lenses (*P*<0.05, Fig. 3 B, right).

Finally, Ndrg2 expression was detected by immunofluorescence staining. As shown in Fig. 3 C, Ndrg2 protein was localized in the cytoplasm of HLECs from both age-related cataract and clear lenses. In the epithelia of cataracts, moderate staining for Ndrg2 was seen in the whole tissues with many strongly stained epithelial cells scattered in them (Fig. 3 C, a–c). In the clear lenses, the immunostaining was moderate or weak (Fig. 3 C, d–f). No staining was observed in negative control cases (data not shown).

## Discussion

NDRG2 has been reported to play important roles in cellular proliferation, differentiation, apoptosis, and response to stress [26]. Up-regulation of NDRG2 in cortical pyramidal neurons and senile plaques of Alzheimer's patients [28], suggested it was associated with ageing. However, Mitchelmore's study reported that NDRG2 up-regulation was associated with Alzheimer's disease rather than specifically associated with ageing. To investigate the potential relationship between NDRG2 and ageing, we employed cultured HLECs exposed to H_2_O_2_ as a model of ageing.

*In vitro* cellular ageing studies have been employed as a model for biological ageing. Exposure to various oxidative stresses, especially sub-lethal doses of H_2_O_2_, has been the most commonly used inducer of premature senescence [36], [37], [38], [39]. Since premature senescence induced by prolonged exposure to oxidative stress shares common mechanisms with pathological ageing *in vivo*, it can serve as a useful *in vitro* model of ageing [40]. To mimic oxidative stress under pathophysiological conditions, SRA01/04 cells were exposed to prolonged low doses of H_2_O_2_, instead of acute high doses of H_2_O_2_, to be induced for ageing. By measuring senescence, DNA synthesis index and activity of SA-β-gal, we found that H_2_O_2_ treated HLECs showed a low ability to synthesis DNA and high SA-β-gal activity, which are the most representative in ageing cells. In addition to that, morphological changes of the cells further confirmed that they are senescent cells. Our model coincides with another ageing model of human diploid fibroblasts that is induced under similar conditions [40]. By using the ageing HLECs, we observed that the expression of Ndrg2 protein markedly increased. To our knowledge, this finding is the first to link NDRG2 expression with cellular ageing.

In order to investigate the possible roles of NDRG2 in HLECs, a NDRG2-overexpression system was established by infecting SRA01/04 cells with Ad-NDRG2. Not as we expected, the overexpression of NDRG2 by itself did not induce senescent biomarkers, such as high SA-β-gal activity, although we observed NDRG2 was up-regulated in senescent HLECs. There are three major alterations in NDRG2 infected cells. Firstly, the cell viability decreased about 20% in NDRG2 infected cells compared with control cells. In addition, NDRG2-infected cells displayed a small but not significant apoptosis (data not shown). This indicates that NDRG2 can decrease the growth and proliferation of HLECs. Our finding is consistent with the studies that NDRG2 reduces the proliferation of many kinds of cancer cells [3], [18], [19], [23], [41], [42]. This suppressed cell proliferation may be through the cellular phase arrest modulated by C-Myc or apoptosis mediated by p53 [42], [43]. Secondly, NDRG2 infected cells showed fibroblast-like features. This is also similar with up regulation of NDRG2 induced morphological changes in malignant breast cancer cells [41]. Moreover, inhibition of differentiation in dendritic cells correlate with reduced NDRG2 expression [21], and NDRG2 in cell differentiation may be regulated by Myc via Miz-1-dependent interaction with the NDRG2 core promoter [44]. Morphological changes of NDRG2 infected cells encourage us to explore its function in differentiation of HLECs.

Last, Ad-NDRG2 infected SRA01/04 cells have lower viability, compared with uninfected and Ad-LacZ infected control cells, when treated with H_2_O_2_. This suggests that up regulation of NDRG2 attenuates the cell resistance to oxidative insult. The lens of the vertebrate eye is a unique organ without blood vessels or innervations. A single layer of lens epithelial cells are essential for maintaining the metabolic homeostasis and transparency of the entire lens. They have the capability of preventing oxidative insult. So we presumed that overexpression of NDRG2 in the cells combined with long term exposure to oxidative stress eventually affect their antioxidative function and lead to cataract formation.

To confirm our presumption, we investigated the expression of NDRG2 in HLECs from age-related cataractous and clear lenses at mRNA and protein levels. Our finding demonstrates that there were about two times of up-regulation of NDRG2 at both mRNA and protein levels in cataract lenses. This implies that NDRG2 is a novel gene that may link to age-related cataract formation.

Although the exact function and mechanism of NDRG2 in the lenses need to be elucidated further, up-regulation of NDRG2 in HLECs not only reduces the cell viability, but also makes HLECs more susceptible to oxidative stress, which induces formation of cataract.

## Materials and Methods

### Cell culture and induction of HLECs ageing

SRA01/04 cells [45] were grown in Dulbecco's modified Eagle's medium (DMEM, GIBCO, Rockville, MD) with 10% fetal bovine serum (FBS, GIBCO, Rockville, MD) at 37°C in an atmosphere of 95% air and 5% CO_2_. Prolonged exposure to H_2_O_2_ to simulate senescence was conduct as previously reported [40]. Briefly, SRA01/04 cells at 80% confluency were treated with H_2_O_2_ at the indicated concentrations (0–200 µM), and incubated at 37°C for 2 weeks. Prolonged H_2_O_2_ exposure was performed by exchanging the old media with fresh H_2_O_2_ (0–200 µM) media that was changed every 3 days over a 2-week period. Parallel cultured control cells were grown in the similar media without H_2_O_2_.

### Ad-NDRG2 infection for NDRG2 overexpression in HLECs

SRA01/04 cells were seeded at densities of 5×10^3^, 5×10^4^, or 2×10^5^ according to the size of the incubation plates (96-well, 24-well or 6-well plates). After 80% confluence, the cells were infected with Ad-NDRG2 (MOI of 50, provided by the Department of Biochemistry and Molecular Biology) for 48 h. Uninfected SRA01/04 cells and cells infected with Ad-LacZ (MOI of 50, provided by the Department of Biochemistry and Molecular Biology) were used as parallel controls.

### Collection of human lens capsule with HLECs

The use of human tissues in this study was in accordance with the Declaration of Helsinki and was approved by the Ethics Committee of the Fourth Military Medical University. The Committee deemed that individual consent was not required from patients as there was no collection of identifying information in association with the samples used. Central pieces of anterior capsules containing attached HLECs from age-related cortical cataracts were collected during cataract surgery. Normal human lenses were obtained from donor eyes for corneal transplantation. The absence of lens opacities in the lenses was confirmed by microscopic examination. Under the stereomicroscope, a corner was removed and the anterior central capsule with attached HLECs was collected. Some capsular samples were rinsed in phosphate buffered saline (PBS), and frozen at −80°C for RT-PCR or Western blot analysis. Others were fixed with 4% paraformaldehyde in PBS for immunofluorescence staining. In all cases of age-related cataract or the clear lenses, persons with other ocular disease, such as glaucoma, uveitis, high myopia, retinal detachment, and trauma, were excluded. Similarly patients with diabetes, malign hypertension, or other systemic diseases were also excluded.

### Induction of acute oxidative stress to NDRG2 overexpression cells

SRA01/04 cells infected with Ad-NDRG2 were exposed to sub-lethal levels of H_2_O_2_ for acute oxidative stress. Forty eight hours after infection, the cells were incubated overnight in DMEM with 2% FBS, followed by serum-free DMEM. After 30 min H_2_O_2_ (50, 100, and 200 µM) was added to the medium and further cultured for 24 h. Uninfected and Ad-LacZ infected SRA01/04 cells served as parallel controls.

### MTT assay

For cell growth and viability assays, SRA01/04 cells were seeded into each well of 96-well plates with 5 replicates for each group at each time point. After stated incubation time, 20 µl MTT solution (5 mg/ml) was added to each well, and after 4 h of incubation, the medium was aspirated as completely as possible without disturbing the formazan crystals. Then, 150 µl DMSO was added to each well and the plates were placed on a plate shaker for 10 min. The OD values at 570 nm were then measured with a Sunrise microplate reader (Tecan, Groedig, Austria).

### SA-β-gal staining

At stated time points, SRA01/04 cells were washed with PBS, fixed in 4% paraformaldehyde for 3–5 min at room temperature, and rinsed with PBS. The cells were then incubated with freshly prepared SA-β-gal stain solution (1 mg/ml X-gal, 40 mM citric acid/sodium phosphate, pH 6.0, 5 mM potassium ferrocyanide, 5 mM potassium ferricyanide, 150 mM NaCl, 2 mM MgCl_2_) overnight at 37°C (without CO_2_). Staining became evident by 2–4 h and was maximal in 12–16 h incubation at 37°C in a CO_2_-free atmosphere. Total and blue stained cells were counted in 10 fields at 100× magnification, and the SA-β-gal-positive cell index was expressed as a percentage of blue stained cells.

### BrdU incorporation assay

Ten µM BrdU was added to SRA01/04 cells treated with 50 µM H_2_O_2_ for 2 weeks. After 1 h of incubation, the cells were fixed with 4% paraformaldehyde for 15 min and immunofluorescence detection of BrdU was performed using the BrdU Labeling and Detection Kit I (Roche Applied Science, Germany) according to the manufacturer's protocol. Nuclei were labeled by counterstaining with DAPI (4, 6′ diamidino-2-phenylinole-2HCI).

### TUNEL assay

Apoptotic SRA01/04 cells were detected by the TUNEL assay using the in situ cell death detection kit (Roche Applied Science, Germany). Nuclei were labeled by counterstaining with DAPI, and the apoptosis index was calculated as the percentage of TUNEL-positive nuclei.

### RNA isolation and Semi-quantitative PCR

Total RNA was extracted from the lens epithelial cells adhering to the anterior capsules using TRIzol Reagent (Invitrogen, Carlsbad, USA) following the manufacturer's instructions. Two µg of total RNA was reverse-transcribed with Oligo-dT primer and reverse transcriptase (Promega, Madison, WI) according to the manufacturer's protocol. The primers for amplifying NDRG2 cDNA were 5′-CCCGGACACAGTTGAAGGT-3′ and 5′- GGGGTCCAGCTTTGAGTTACA TT-3′, which amplify cDNAs of 310 bp. The indicated ACTIN control reactions were also performed. The sequence of ACTIN primers were: 5′-TTCGTGGATGCCACAGGACT-3′ and 5′-TCACCAACTGGGACGACATG-3′, and the PCR product is 602 bp. RNA samples from pools of 30 age-related cortical cataractous and 10 clear anterior capsules were analyzed by PCR. Taq DNA polymerase (Promega, Madison, WI) dependent DNA amplification and reactions were set up based on the manufacture's protocol. The PCR reactions were initiated with denaturation at 95°C for 5 min, followed by 25 cycles of thermal cycling (30 s at 94°C, 40 s at 55°C and 60 s at 72°C). The final cycle was followed by 7 min of an extension step at 7°C. The reaction parameters were adjusted so that a linear relation between the number of PCR cycles and PCR products, and a linear relation between the initial amount of cDNA template and PCR products were obtained. Products were subsequently separated on 1.2% agarose gels and visualized by ethidium bromide staining. The results were normalized using the ratio of the band density of NRDG2 mRNA to ACTIN mRNA. With the other 60 age-related cortical cataracts and 20 clear lens capsular epithelial cell samples prepared for RT-PCR study, the analysis was repeated two more times, respectively, initiated with different tissue pools. The results were then statistically analyzed.

### Protein extraction and Western blot analysis

Cultured cells or anterior capsules adhering with lens epithelial cells were extracted in lysis buffer ( 20 mM Tris, pH 7.4, 150 mM NaCl, 1% Triton X-100, 10% glycerol, 1.5 mM MgCl_2_, 1 mM EDTA, 1 mM phenylmethylsulfonyl fluoride, 0.5 mg/ml leupeptin, and 1 mg/ml aprotinin). Protein concentrations were determined by the bicinchoninic acid (BCA) assay. Equal amounts of total cellular proteins (15 µg) were seperated on 10% SDS-polyacrylamide gels, and electrophoretically transferred to a nitrocellulose membrane. The blots were probed with anti-Ndrg2 goat polyclonal antibody (Santa Cruz, CA). Equal loading of all lanes was confirmed by reprobing the membrane with anti-Tubulin mouse monoclonal antibody (Santa Cruz, CA). Horseradish peroxidase-conjugated secondary antibodies were obtained from Jackson ImmunoResearch Labratories (West Grove, PA). Densitometric analysis was performed using Kodak Digital Science 1D software (Kodak, New Haven, CT). The experiments were repeated two more times, respectively, with different tissue or the cell pools. The results were then statistically analyzed.

### Histological Immunofluorescence staining

Since the lens epithelium is a monlayer of cells that adhere to the transparent capsule, cellular immunostaining can be directly done by placing the capsule in a small well. Ten anterior capsules from age-related cataracts were used for immunofluorescence staining and 5 capsules from the clear lenses were used as controls. The fixed capsules were washed with PBS and blocked with buffer containing 1∶50 normal goat serum and 1% bovine serum albumin for 1 h. The capsules were then incubated with primary monoclonal Ndrg2 antibody (FMU-Ndrg2.3), which was obtained from mouse immunized with recombinant His-tag human Ndrg2 protein, and shown specifically to react with human and mouse Ndrg2 [46]. This was followed by a fluorescein isothiocyanate (FITC) conjugated secondary antibody. Cell distribution was visualized by staining with DAPI. After washing, the capsules were mounted onto micro slides. Negative controls were similarly prepared except that the primary antibody was replaced with PBS, normal serum, or mouse isotype IgG1. Fluorescence stained samples were examined with a laser scanning confocal microscope (FV500IX-70, Olympus, Japan). All images were captured under the same condition.

### Statistical analysis

Statistical analysis was performed with the SPSS 12.0 program. All data from quantitative assays were expressed as Mean± SD. Comparisons between groups were undertaken using Student's *t*-test and one-way ANOVA analysis. *P*<0.05 was considered statistically significant.
